# Distinct Variations in Gene Expression and Cell Composition across Lichen Planus Subtypes

**DOI:** 10.3390/ijms25179720

**Published:** 2024-09-08

**Authors:** Cadri Knoch, Veronika Baghin, Patrick Turko, Nicola Winkelbeiner, Ramon Staeger, Kongchang Wei, Irina Banzola, Mark Mellett, Mitchell P. Levesque, Thomas Kuendig, Lars E. French, Lucie Heinzerling, Barbara Meier-Schiesser

**Affiliations:** 1 Department of Dermatology, University Hospital Zurich, 8091 Zurich, Switzerland; cadriisabelle.knoch@usz.ch (C.K.); veronika.baghin@usz.ch (V.B.);; 2 Empa, Swiss Federal Laboratories for Materials Science and Technology, Laboratory for Biomimetic Membranes and Textiles, Laboratory for Biointerfaces, 9014 St. Gallen, Switzerland; 3 Department of Urology, University Hospital Zurich, 8091 Zurich, Switzerland; 4 Department of Dermatology and Allergy, University Hospital, LMU Munich, 80337 Munich, Germany; 5 Department of Dermatology and Cutaneous Surgery, University of Miami Miller School of Medicine, Miami, FL 33136, USA; 6 Department of Dermatology and Allergy, University Hospital Erlangen, 80337 Erlangen, Germany

**Keywords:** lichen planus, lichen planus subtypes, NanoString, multiplex immunohistochemistry, immune cell infiltrate

## Abstract

Lichen planus (LP) is a highly prevalent inflammatory skin disease. While various clinical subtypes have been defined, detailed comparisons of these variants are lacking. This study aimed to elucidate differences in gene expression and cellular composition across LP subtypes. Lesional skin biopsies from 28 LP patients (classical, oral, genital, and lichen planopilaris) and seven non-diseased skin controls (NDC) were analyzed. Gene expression profiling of 730 inflammation-related genes was conducted using NanoString. Immune cell compositions were assessed by multiplex immunohistochemistry. Gene expression profiles revealed unique inflammatory signatures for each LP subtype. Lichen planopilaris exhibited the most divergence, with downregulated gene expression and upregulation of complement pathway genes (*C5-7*), along with elevated M2 macrophages. Oral and genital LP demonstrated similar profiles with strong upregulation of TNF-related and Toll-like receptor-associated genes. Oral LP showed the highest upregulation of cytotoxicity-associated genes, as well as high numbers of CD8+ IL-17A+ (Tc17) cells (8.02%). Interferon gene signatures were strongly upregulated in oral and classical LP. The study highlights distinct differences in inflammatory gene expression and cell composition across LP subtypes, emphasizing the need for tailored therapeutic approaches.

## 1. Introduction

Lichen planus (LP) is an inflammatory disease of high prevalence affecting the skin, mucous membranes, the hair, and nails. It is one of the most common inflammatory skin diseases, with a prevalence of 0.1–1.2% [1,2] of the worldwide population.

Several subtypes have been described based on the morphology of the lesions and the site of involvement: the classical form, a hypertrophic form, a mucosal form (oral or genital LP), LP of the scalp (lichen planopilaris), LP of the nails, and several other rare forms [3]. LP can potentially cause severe and disabling symptoms that have a significant impact on patients’ lives, particularly of those with mucosal LP. Moreover, both mucosal and cutaneous LP are considered potential premalignant conditions [3,4]. The exact pathomechanism of LP remains unclear, which is reflected by the lack of specific and potent treatments, as well as by the inability to predict a patient’s specific response to available therapies. Cell-mediated cytotoxicity seems to play a major role in lichenoid skin inflammation, as there is dense infiltration of both CD4+ and CD8+ cells and keratinocyte apoptosis [5]. Even though the T-cell infiltrate, the immune repertoire, and some genetic and environmental factors were partially characterized in some forms of LP [5,6,7], to date no groundbreaking insights into its pathogenesis could be found. Only recently, IFNγ has been identified as enhancer of T-cell-mediated cytotoxic responses against keratinocytes in classical and hypertrophic LP, and this was dependent on the JAK2/STAT1 pathway [8]. This could partially be translated into clinical disease management as individual cases respond to JAK inhibition [9,10,11]. Furthermore, new data points towards a potential importance of Th17 cells in classical and oral LP [12], which is supported by the fact that certain LP patients respond well to a treatment with secukinumab (anti-IL-17), ustekinumab (anti-IL-12/23), or guselkumab (anti-IL-23) [13].

However, current research has largely treated LP as a monolithic entity with insufficient differentiation between these subtypes. This approach limits the ability to develop tailored therapeutic strategies that consider the specificities of each subtype, potentially impacting patient outcomes. The aim of this study was to thoroughly examine and identify the differences among the various subtypes of lichen planus (LP). By focusing on the distinct characteristics that define each subtype, the study seeks to gain insights that can guide the development of more precise and effective therapeutic strategies tailored to the specific needs of each LP subtype.

## 2. Results

### 2.1. Clinically Known LP Subtypes Show Distinct Gene Expression Patterns

**Figure 1:**
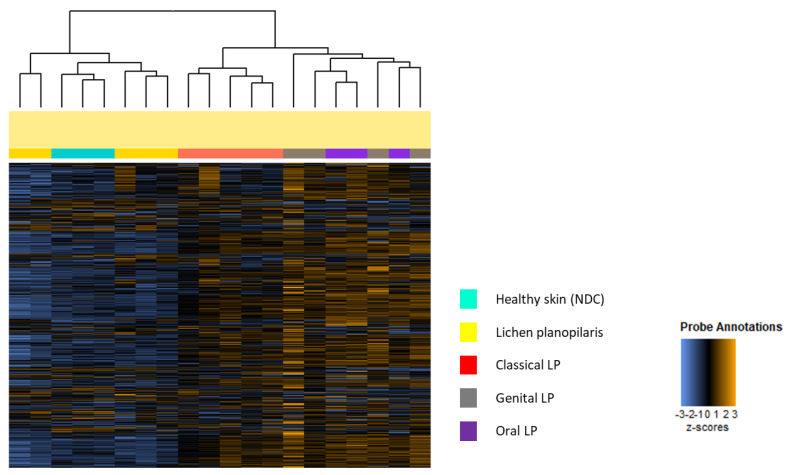
Clear distinction of different lichen planus subtypes and healthy skin in gene expression analysis. NanoString technology was applied to compare the expression of 730 inflammation-related genes across lesional skin samples from 5 classical lichen planus (LP), 4 genital LP, 3 oral LP, and 5 lichen planopilaris patients and 3 healthy skin (NDC) samples. Heatmap showing hierarchical clustering of different LP subtypes and NDC.

To determine possible differences in the gene expression pattern of different LP subtypes, a NanoString analysis of 730 inflammation-related genes was performed on lesional LP skin and healthy skin (non-diseased control = NDC) as a control. Notably, the clinically known LP classical, oral, genital, and lichen planopilaris and NDC showed distinct clustering. Interestingly, lichen planopilaris clustered with NDC, while the mucosal LP forms cluster together and display the most significant gene upregulation (Figure 1). The highest inflammatory gene expression was observed in oral LP, with decreasing levels noted in genital, classical, and lichen planopilaris subtypes (Supplementary Figure S1A–D).

In the direct comparison of oral LP and NDC, 135 genes with significant differential expression were identified. The upregulated genes were predominantly linked to cytotoxicity, cancer/testis (ct) antigen, and the complement system (Supplementary Figure S1A, Supplementary Table S1A). For genital LP versus NDC, 86 genes with significant changes in expression were noted. Prominent among the upregulated genes were those associated with Toll-like receptors (TLRs), microglial function, and B-cell-functions (Supplementary Figure S1B, Supplementary Table S1B). When examining classical LP relative to NDC, 54 genes demonstrated significant differential expression. Cytotoxicity, ct antigen, and regulatory functions were key among the upregulated gene sets (Supplementary Figure S1C, Supplementary Table S1C). When comparing lichen planopilaris and NDC, the findings revealed only 16 genes with significant differential expression. Among these, the most prominently affected genes were related to ct antigen and regulatory function (Supplementary Figure S1D, Supplementary Table S1D).

In addition to comparing LP subtypes with NDC, the LP groups were also compared among themselves. Comparing genital and oral LP, 68 genes were significantly differentially expressed. Key affected genes were linked to ct antigen, cell cycle, and adhesion (Supplementary Figure S1E, Supplementary Table S1E). In the comparison between classical and genital LP, only 19 genes showed significant differences. The most affected genes were related to TLR signaling, microglial function, and adhesion (Supplementary Figure S1F, Supplementary Table S1F). Comparing classical and oral LP, 36 genes showed significant differential expression. Notably, the most impacted genes pertained to interleukins, pathogen defense, and macrophage function (Supplementary Figure S1G, Supplementary Table S1G). In the classical versus lichen planopilaris comparison, 134 genes were differentially expressed. The most significantly affected genes were related to cytotoxicity, antigen processing, and T-cell function (Supplementary Figure S1H, Supplementary Table S1H).

### 2.2. LP Subtypes Exhibit Pathway-Specific Differences in Gene Expression

**Figure 2:**
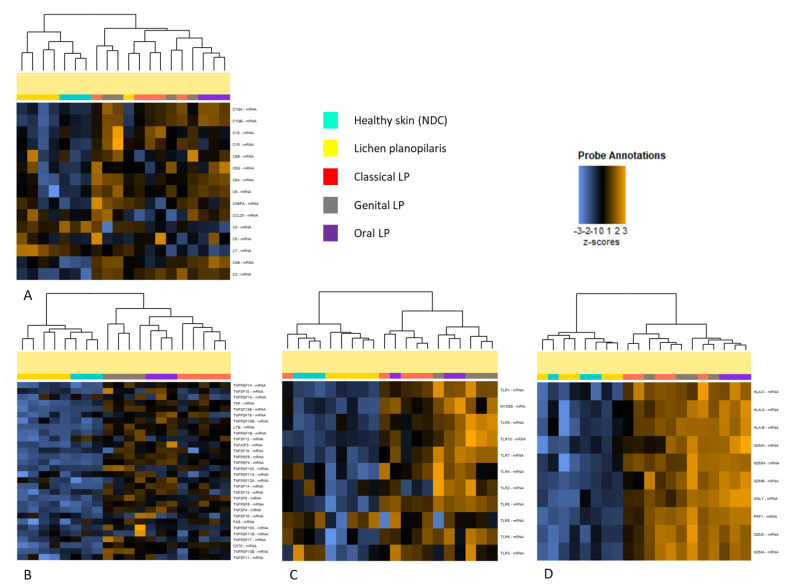
Pathway-specific differences in gene expression across different lichen planus subtypes. NanoString technology was applied to compare the expression of different immune−related genes across lesional skin samples from 5 classical lichen planus (LP), 4 genital LP, 3 oral LP, and 5 lichen planopilaris patients and 3 healthy skin samples. Heatmaps showing genes related to complement pathway (**A**), TNF−related genes (**B**), Toll−like receptor (TLR)−related genes (**C**), and cytotoxicity−related genes (**D**).

To detect pathway-specific differences between LP subtypes, a gene-set analysis was performed. Like in the overall gene expression pattern, lichen planopilaris is distinctly separated from other LP subtypes. While gene expression is mostly downregulated in lichen planopilaris, selected genes of the complement pathway (*C5-7*) were upregulated in lichen planopilaris compared to the other subtypes and NDC (Figure 2A). When analyzing TNF-related genes such as *TNF*, *LTB*, *FAS*, and *CD70*, a distinct clustering of the different LP subtypes was noticeable with the highest expression in both mucosal LP subtypes (oral and genital LP) (Figure 2B). Genes associated with cytotoxicity and TLR were strongly upregulated in both mucosal and classical lichen subtypes. Notably, oral LP demonstrated the highest upregulation of various cytotoxicity-associated genes, such as *HLA-A/B/C*, *PRF1* (Perforin), *GZMB* (Granzyme B), and *GNLY* (Granulysin) (Figure 2D). Genes associated with TLRs showed the highest upregulation in oral and genital LP (Figure 2C).

Further analysis of gene expression patterns across various LP subtypes identified a pronounced interferon gene signature specifically in the oral and classical LP variants. This signature was marked by significant upregulation of *MX1* (fold change = 10.57, *p* = 0.0038), *CXCL9* (fold change = 51.19, *p* = 0.0036), *IRF8* (fold change = 6.67, *p* = 0.0027), and *IRF7* (fold change = 8.80, *p* = 0.0005) in oral LP compared to NDC. In classical LP, there was a notable increase in the expression of *CXCL9* (fold change = 31.62, *p* = 0.04) and *IRF3* (fold change = 2.27, *p* = 0.0045). In contrast, the genital and lichen planopilaris variants showed no enhanced expression of interferon-related genes. Additionally, gene expression analysis revealed upregulation of genes associated with neutrophil granulocytes (*CSF3R* fold change = 2.42, *p* = 0.005; *CXCL8* fold change = 10.08, *p* = 0.0067) exclusively in genital LP.

### 2.3. Variations in Cellular Compositions Distinguish Clinical LP Subtypes

**Figure 3:**
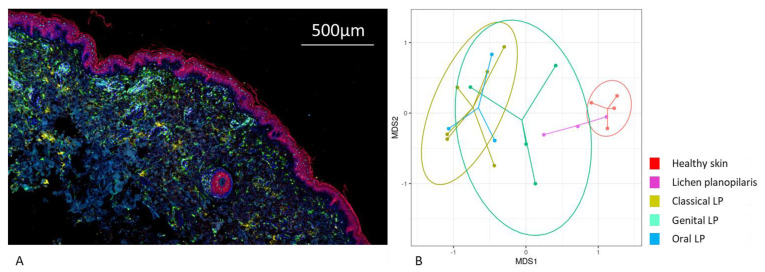
Multiplex immunohistochemical analysis reveals different cell-type compositions among lichen planus subtypes. Cellular composition among different subtypes of lichen planus (LP) was investigated by multiplex immunohistochemistry using the Akoya system. Two different antibody panels (T-cell panel: CD8, FoxP3, IL17A, Granzyme B, PanCK, CD4; macrophage panel: pSTAT1, MPO, c−Maf, PanCK, CD68) were developed for staining. (**A**) Visualization by application of Opal fluorescent dyes. (**B**) The non−metric multidimensional scaling (NMDS) visualization highlights the intrinsic differences in cell-type composition between the various subtypes of LP.

To discriminate LP subtypes based on cell-type composition, a multiplex immunohistochemical staining using the Akoya system was performed on lesional skin of classical, oral, genital, and lichen planopilaris patients, as well as on NDC. Stainings were performed using antibodies for the identification of CD4+ and CD8+ T cells, regulatory T cells (Tregs), Th17 cells, the cytotoxicity marker Granzyme B, macrophages (M1 and M2), neutrophilic granulocytes, and keratinocytes (representative staining Figure 3A).

To gain insight into the uncombined overall cell-type distinctions between the LP subtypes, inter-sample multivariate “distance” scores were calculated using the Bray–Curtis dissimilarity index and distances were visualized using non-metric multi-dimensional scaling (NMDS, Figure 3B). We tested for overall differences among the subtypes with PERMANOVA, with highly significant results (*p* = 0.002). Next, to investigate pairwise differences in cell type composition between each of the LP subtypes, we performed a PERMANOVA between each pair of LP subtypes. These comparisons (Supplementary Figure S2) demonstrated that all LP subtypes—except lichen planopilaris—significantly differ in their cellular composition from NDC. However, among the LP subtypes, the cellular compositions differed only significantly between classical LP and lichen planopilaris (*p* = 0.01).

These pairwise dissimilarities between subtypes (Supplementary Figure S3) were further decomposed into contributions from each cell type using SIMPER analysis. Given an overall dissimilarity between LP subtypes, SIMPER gives further insight into how much of that dissimilarity can be explained by each cell type. Thus, when decomposing each LP subtype’s dissimilarity from healthy dermal skin, we see that the differences are mostly driven by the overall greater prevalence of immune cells in LP skin. This effect accounts for between 19% and 33% of the dissimilarities.

In detail, classical LP dissimilarity from NDC is driven by Tregs (36% of total dissimilarity, *p* = 0.004) and macrophages (12%, *p* = 0.01), with small but significant contributions by CD4+ and CD8+ T cells (*p* = 0.03, 0.002, respectively). Scalp vs. NDC dissimilarity is driven by Tregs (5%, *p* = 0.9) and macrophages (6%, *p* = 0.7). Genital vs. NDC dissimilarity is driven by Tregs (19%, *p* = 0.7), macrophages (6%, *p* = 0.6), CD4+ T cells (6%, *p* = 0.3), and CD8+ T cells (5%, *p* = 0.3). Oral vs. NDC dissimilarity is driven by Tregs (36%, *p* = 0.02), IL17A cells (8%, *p* < 0.001), and keratinocytes (8%, *p* < 0.001). In all cases, consistency of samples determined the *p*-value associated with these percentages.

Finally, the dissimilarity between classical LP and lichen planopilaris is driven by overall immune infiltration in classical LP. This effect accounts for 29% of the dissimilarity (*p* = 0.09), with the next most important cell type being Tregs (28%, *p* = 0.1).

**Figure 4:**
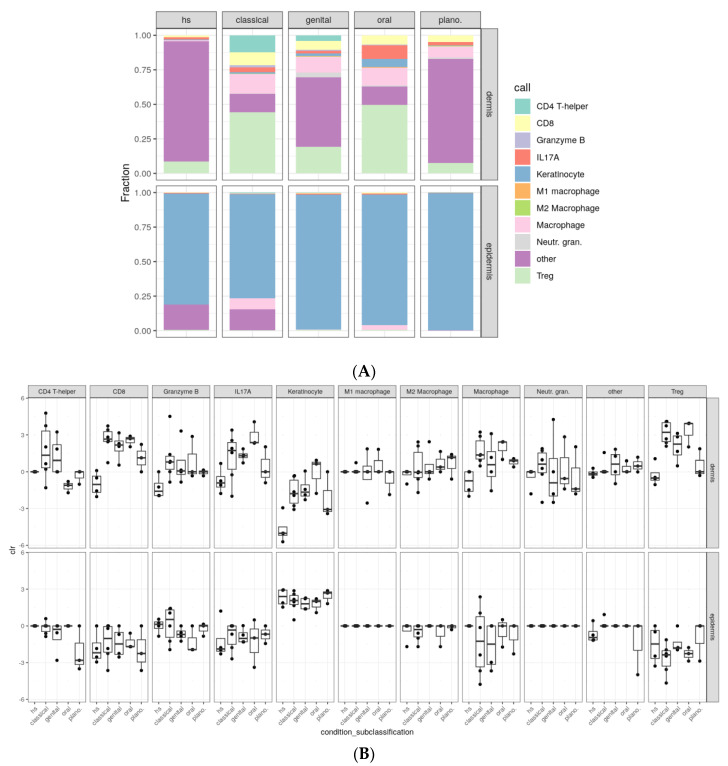

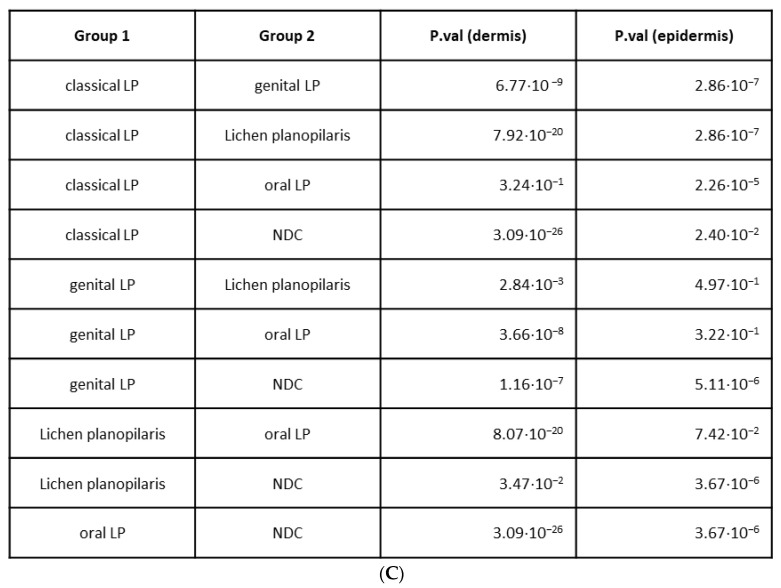
Significant differences in cell-type composition across different lichen planus subtypes. Cellular composition across different lichen planus (LP) subtypes, including 4 cases of healthy skin, 6 cases of classical LP, 4 cases of genital LP, 3 cases of oral LP, and 3 cases of lichen planopilaris, was assessed by multiplex immunohistochemistry using the Akoya system. The frequency of CD4+ T cells, CD8+ T cells, regulatory T cells (Tregs), cytotoxic cells (Granzyme B+), Th17 cells (IL−17A), keratinocytes, M1 and M2 macrophages, total macrophages, neutrophilic granulocytes, and other cells was assessed. (**A**) shows the cell−type composition of all samples, (**B**) represents individual testing of cell types in the dermis, with data being transformed using the centered−log−ratio transformation. (**C**) Statistical analysis of differences between the groups was performed using Fisher’s exact test.

Next, a detailed analysis of the cell type composition was performed. Combining all dermal cells from each subtype (Figure 4A) and testing inter-type differences with Fisher’s exact test, we see that almost all subtypes significantly differ from each other (Figure 4C).

Even though the overall cell type analysis of the dermis of lichen planopilaris closely resembled that of NDC, the individual testing of cell types revealed elevated levels of CD8+ T cells (2.17%) compared to NDC dermis (0.373%). In contrast, the dermis of lichen planopilaris was characterized by a deficiency in CD4+T cells and a less pronounced presence of Tregs (3.51%) compared to the dermis of other LP subtypes. Further, the dermis of lichen planopilaris showed a lower presence of IL-17A-producing cells (1.18%) compared to oral LP (8.02%) but not compared to the other examined LP subtypes (Table 1).

The dermal infiltrate of classical LP exhibited higher numbers of CD4+ T cells (8.50%), CD8+ T cells (6.44%), Tregs (30.3%), and macrophages (12.3%) compared to the dermis of NDC (CD4+ T-cells 0%; CD8+ T-cells 0.373%; Tregs 2.29%; and macrophages 0.193%) (Table 1). Numbers of CD4+ T cells, CD8+ T cells, Tregs, macrophages, and neutrophilic granulocytes were also higher in genital LP (CD4+ T-cells 3.14%, CD8+ T-cell 4.87%, Tregs 14.6% and macrophages 9.62%) compared to NDC (Table 1) and this was supported by high sample distances in the Bray–Curtis dissimilarity matrix (Supplementary Figure S3).

In oral LP, the dermis demonstrated a higher presence of both CD8+ T cells (5.27%) and Tregs (39.2%) compared to the dermis of NDC. Moreover, there was a higher number of IL-17A-producing cells (8.02%) in oral LP compared to NDC (0.831%). The number of IL-17A-producing cells was also the highest in oral LP compared to all other LP subtypes (classical LP 2.39%, genital LP 1.54%, lichen planopilaris 1.18%; Table 1). The fact that CD4+ T cells showed increased amounts only in the dermis of classical and genital LP is suggestive of CD8+ T cells being the main producers of IL-17A in oral LP.

All LP subtypes except for genital LP demonstrated significantly elevated levels of total macrophages in comparison to NDC (classical LP 12.3%, genital LP 8.82%, oral LP 10.8%, lichen planopilaris 3.74%, NDC 0.193%). Notably, a detailed analysis of M1 and M2 macrophage subtypes revealed higher amounts of M1 macrophages in all LP subtypes (classical 0.0397%, genital LP 0.243%, oral LP 0.266%, lichen planopilaris 0.0092%) compared to NDC (0.0%), while M2 macrophages were particularly increased in lichen planopilaris (0.212%) compared to NDC (0.0126%) (Table 1; Figure 4B).

## 3. Discussion

LP subtypes exhibit consistent histological characteristics; however, their clinical manifestations can vary significantly [1]. Although recent studies have sought to elucidate the pathomechanisms underlying LP, the distinctions among LP subtypes have not been adequately explored. Here we studied the differences in inflammatory gene expression and cell-type composition among classical, oral, and genital LP and lichen planopilaris and NDC as control. NanoString analysis of 730 genes associated with inflammation demonstrated distinct clustering of samples corresponding to the same LP subtype, suggesting that different LP subtypes may represent unique pathophysiological entities.

Lichen planopilaris exhibited significant differences from other LP subtypes in terms of gene expression and cellular composition. This divergence is mirrored in its clinical presentation, which shares few similarities with the cutaneous and mucosal forms of LP. The low levels of inflammation-related genes and the marginal inflammatory cell infiltrate might further partly be attributed to the time point of the biopsy as this is mostly taking place when scarring has occurred. The M2 macrophages were elevated when compared to other LP subtypes and NDC. This might be explained by the fact that lichen of the scalp is a scarring disease which is related to the appearance of M2 macrophages [14]. However, also compared to another scarring disease of the scalp, frontal fibrosing alopecia, an increased M2 phenotype in lesional skin of lichen planopilaris was found in a study from Kinoshita-Ise [15]. A direct comparison of macrophage polarization between lichen planopilaris and other LP subtypes has not been reported to date. Our data points towards a potential importance of macrophage polarization in lichen of the scalp; however, broader and functional studies are needed. Moreover, while most genes were downregulated compared to the other LP subtypes, the complement factors C5, C6, and C7 were upregulated on the mRNA transcript level. To our best knowledge, an impact of the complement cascade has not been reported to date and is of interest for further investigation.

Notably, when looking at specific pathway-related genes, we observed the highest upregulation of several cytotoxicity-associated genes in oral LP, which reflects the aggressive nature of mucosal LP and frequent development of erosions in oral LP. Our observation that a pronounced interferon gene signature was specifically detected in the oral and classical LP variants is partly supported by other studies showing that the inflammation in cutaneous and oral LP is dominated by IFNγ, amongst others [16]. Specific for classical LP was the strong upregulation of *CXCL9* and *IRF3*. Interestingly, Wenzel et al. have shown that CXCL9-mediated inflammation distinguished LP from atopic dermatitis and psoriasis [17]. Surprisingly, interferon-related genes were not upregulated in genital LP. Indeed, an important role of interferons has not been reported to date in genital LP; however, several cases of erosive vulvovaginal LP have been successfully treated with the JAK inhibitor tofacitinib, thereby also targeting interferon signaling [18].

Our finding that IL-17A-positive cells were higher in oral LP than in other LP subtypes is suggestive of an involvement of Th17 cells. This is supported by data from Miyahara et al. as it has been shown that epithelium-derived cathepsin K can stimulate Toll-like receptor 9-positive plasmacytoid dendritic cells to promote Th17 immune responses in oral lichen planus [19]. Another recent study has demonstrated that an NF-κB-driven upregulation of renin promotes IL-17 expression in oral keratinocytes of LP [20]. As the wide majority of oral T cells were CD8-positive in our stainings, we hypothesize that IL-17+ CD8+ T cells (Tc17) are the main producers of IL-17A in oral LP and might play a key role in the pathogenesis of LP. These findings are also clinically relevant as certain patients responded well to a treatment with secukinumab (anti-IL-17), ustekinumab (anti-IL-12/23), or guselkumab (anti-IL-23), but this was not limited to oral LP [12,13].

Tregs represent one of the cell populations that were mainly responsible for the strong differences in cell type composition. They were particularly high in classical and oral LP. This is in line with the literature as Tregs have been found in high numbers in cutaneous and even more in oral LP [21,22,23,24]. The exact role of T regs in the pathogenesis of LP has not been elucidated to date, but one study showed that CD4+CD25+ cells in oral LP were increased but functionally impaired [25]. Further mechanistical studies are needed to understand the impact of Tregs in disease development or maintenance.

Furthermore, genes linked to the TNF superfamily were significantly upregulated in both forms of mucosal LP but not in other studied subtypes. Although several case reports have shown successful treatment outcomes [21,22,23], broader studies on this subject are lacking. To our best knowledge, this is the first report indicating that TNF cytokines are predominantly present in mucosal LP. Additionally, TLR-related genes showed the highest upregulation in both mucosal LP forms. These findings suggest a possible role of the innate immune system in both oral and genital LP. Interestingly, a study by Carvalho et al. showed an enhanced TNF response for extracellular TLR stimulation in LP compared to healthy peripheral mononuclear cells [26] and are of potential interest for future therapies. Moreover, the elevation of TLR genes might be indicative of preceding infections in mucosal LP as TLRs play an important role in immune responses to bacterial and/or viral infections [27]. Indeed, associations between oral LP and hepatitis C [28,29] but also other viral infections have been reported [30]. Further supportive of a potentially important role of innate immunity in genital LP is the fact that genes associated with neutrophil granulocytes were strongly upregulated in this LP subtype in our analysis.

Limitations of our study include the small sample size with a consequential low statistical power in the individual testings of cell types, leading to less significant results than expected from raw percentages, as well as the lack of a functional proof of our findings and suggested mechanisms.

Collectively, our study showed distinct differences in inflammatory-related gene expression and cell composition across LP subtypes. Further studies focusing on the pathomechanisms and response to treatment among the subtypes of LP are crucial to enhance the precision of care in clinical settings.

## 4. Materials and Methods

### 4.1. Human Skin Samples

Lesional skin biopsies from 28 LP patients and non-diseased skin of seven healthy donors were collected at the dermatology department of the University Hospital of Zurich and included in this study. All patients had a clinical and histopathological matching diagnosis of a LP subtype. In detail, eight classical (cutaneous), five oral, and seven genital LP and seven lichen planopilaris samples were included. Patients who had received topical or systemic steroid treatments within two weeks prior to the biopsy were excluded from the study. In addition, none of the patients had ever received any kind of immune-modulating or immunosuppressive therapy. All patients have signed the informed consent, this project was approved by the Cantonal Ethics Committee of the Canton of Zurich, Switzerland (approval no. 2021-00958). Patients’ characteristics are presented in Table 2.

All patient information was analyzed after informed, written patient consent. The study had the approval of the local ethics committees and was carried out according to the Declaration of Helsinki Principles (BASEC 2021-00951).

### 4.2. NanoString Analyses

RNA was extracted using Trizol, followed by RNA purification steps using the RNeasy FFPE Kit (Qiagen, Hilden, Germany) according to manufacturer’s instructions. The gene expression analysis was performed on NanoString for 730 genes. Expression was determined using the nCounter PanCancer Immune Profiling Panel™ (human) (XT-CSO-HIP1-12, NanoString, Seattle, WA, USA). Briefly, 200–400 ng of RNA was applied for the sample hybridization, which was performed according to the manufacturer’s protocol. The nCounter^®^ Flex Digital analyzer (NanoString, Seattle, WA, USA) was used for sample detection and analysis.

The raw data processing, quality control, and normalization were run on the ROSALIND^®^ analysis software (Version 3.35.3.0; San Diego, CA, USA https://rosalind.onramp.bio/ accessed on 1 April 2024) as previously described [31], as well as on the nSolver^®^ software (Version 4.0, NanoString, Seattle, WA, USA).

### 4.3. Multiplex Immunohistochemistry

Two multiplex panels were developed for immunohistochemical staining of T cells and inflammatory cytokines (T-cell panel) and macrophages and neutrophils (Macrophage panel) on 3 µm paraffin-embedded sections.

Staining was automated using a Bond RXm autostainer (Leica Biosystems, Nussloch, Germany) and OPAL 7-Color Automation IHC Kit (Akoya Biosciences, Marlborough, MA, USA). The process included deparaffinization, multiple washes, antigen retrieval with pH6 buffer and blocking with Opal Antibody Diluent/Block (Akoya Biosciences, Marlborough, MA, USA). Stainings with primary antibodies (T-cell panel: CD8, FoxP3, IL17A, Granzyme B, PanCK, CD4; macrophage panel: pSTAT1, MPO, c-Maf, PanCK, CD68) were performed sequentially; dilutions are specified in Table 3. DAPI served as a counterstain.

After primary antibody incubation, sections underwent further washing and incubation with Opal Polymer HRP Ms + Rb, followed by application of Opal fluorescent dyes, diluted as specified. The Tyramide Signal Amplification method enhanced sensitivity and enabled multicolor staining. For non-standard Opal 780, additional steps, including TSA-DIG incubation and extra washing, were added. After mounting, stained slides were scanned with an AKOYA PhenoImager HT 2.0.

### 4.4. Statistical Analysis

NanoString data were analyzed using the fast method (nCounter Advanced Analysis 2.0 User Manual) for calculating fold changes and *p* values, with *p* values adjusted via the Benjamini–Hochberg method. Cut-offs were established at a fold change of 1.5 and a *p* value adjustment of ≤0.05.

Akoya staining images were segmented using adaptive segmentation in InForm version 2.6.0 (Akoya Biosciences). Nuclei were detected using the DAPI channel, and cytoplasmic segments were assumed to form non-overlapping rings around each nucleus. Mean staining intensity for each cellular compartment was output.

These cellular intensity measurements were used to assign each cell a state of “positive” or “negative” separately for each marker by fitting additive mixture models to intensity histograms using the R package mixR [32]. Histograms were transformed using the hyperbolic arcsin transformation when required to obtain accurate model fits, and, depending on the shape of the histogram, we fit either normal, log-normal, or Weibull distributions. We then calculated the probability of belonging to the “positive” distribution for each cell and assigned this state when *p* > 0.95. Finally, each cell was assigned a composite phenotype based on its marker positivity. Cells negative for all markers were assigned the phenotype “other”.

Compositional differences were tested in two ways. First, all cells from all samples of the same subtype were combined into one composition, and differences between these “overall” compositions were analyzed using Fisher’s exact test. Comparisons are made separately for the dermis and the epidermis. Second, to better account for inter-subtype heterogeneity, we performed an ordination analysis common to community ecology. An inter-sample multi-variate distance matrix was constructed using the Bray–Curtis dissimilarity. These dissimilarities were visualized using non-metric multi-dimensional scaling (NMDS). We tested the significance on inter-group dissimilarities using permutational multivariate ANOVA (PERMANOVA). Finally, the contribution of each cell-type to the inter-subtype dissimilarities was calculated using the “similarity percentages” method (“simper”). Simper analysis assigns each cell type a magnitude of how strongly it affects the inter-subtype compositional dissimilarity and a *p*-value according to the consistency of the samples. All ordination analyses were performed using the R package “vegan” [33].

Differences in the prevalence of individual cell types between LP subtypes were tested using ANOVA after transformation of cell-type percentages to account for their compositional nature with the centered log-ratio transformation (“CLR”) [34].

## Figures and Tables

**Table 1:** Raw percentages of cell types in lichen planus subtypes and healthy skin.

Cell Types	Classical LP	Genital LP	Oral LP	Lichen Planopilaris	NDC
Total T cells	48.8%	24.7%	53.0%	7.04%	3.61%
CD4 + T cells	8.5%	3.14%	0.108%	0.069%	0%
CD8 + T cells	6.44%	4.87%	5.27%	2.17%	0.373%
Tregs	30.3%	14.6%	39.2%	3.51%	2.29%
Granzyme B+ cells	1.15%	0.619%	0.434%	0.110%	0.117%
IL17A+ cells	2.39%	1.54%	8.02%	1.18%	0.831%
Keratinocytes	24.9%	25.4%	24.6%	54.0%	62.1%
Total macrophages	12.3%	9.26%	11.2%	3.96%	0.193%
M1 macrophages	0.0397%	0.243%	0.266%	0.0092%	0%
M2 macrophages	0.176%	0.191%	0.124%	0.212%	0.0126%
Neutrophilic granulocytes	0.301%	2.51%	0.730%	0.446%	0.0084%
Other	13.8%	38.1%	10.5%	34.6%	34.1%

**Table 2:** Patient characteristics.

Characteristics of Patients		Number of Patients, No.	Age y, Mean (SD)	Female/Male Sex, Ratio	Other Autoimmune Disease, No.	HBV or HCV Infection, No.	Topical Therapy at Time of Biopsy *, No.	Disease Onset Until Biopsy Months, Mean (SD) **
Classical LP	NanoString	5	64.0 (13.2)	2/3	0	0	0	4 (1.8)
	IHC	6	65.0 (15.3)	3/3	0	0	0	10 (14.8)
Oral LP	NanoString	3	49.0 (8.9)	1/2	0	0	0	2 (0.6)
	IHC	3	52.0 (6.6)	1/2	0	0	0	11 (5.7)
Genital LP	NanoString	4	59.8 (17.4)	0/4	0	1	1	14 (14.1)
	IHC	4	56.5 (19.6)	3/1	0	0	0	11 (2.7)
Lichen planopilaris	NanoString	5	56.2 (17.6)	5/0	2	0	2	24 (14.4)
	IHC	3	45.7 (17.5)	2/1	1	0	0	24 (20.2)
Healthy skin (NDC)	NanoString	3	81.0 (7.9)	1/2	0	0	0	0
	IHC	4	57.0 (20.4)	0/4	0	0	0	0

Nanostring: samples analyzed using NanoString technology (NanoString, Seattle, WA, USA); IHC: samples analyzed with multiplex immunohistochemistry. HBV = hepatitis B virus, HCV = hepatitis C virus. * The topical therapy included Dalacin solution (Clindamycin 10 mg/g) and moisturizers. ** Values were capped at 36 months for this aggregation.

**Table 3:** Dilution of antibodies and assigned Opal fluorophores.

Opal	T-Cell Panel		Macrophage Panel	
	Antibody	Dilution	Antibody	Dilution
480	CD8	1:200	pSTAT1	1:50
520	FoxP3	1:100		
570	IL17A	1:200	MPO	1:150
620	GranzymeB	1:100	c-Maf	1:50
690	PanCK	1:100	PanCK	1:100
780	CD4	1:100	CD68	1:100

## Data Availability

The data presented in this study are available on request from the corresponding author.

## Supplementary Materials

The following supporting information can be downloaded at: https://www.mdpi.com/article/10.3390/ijms25179720/s1.
