# Supplementary material for: Distinct Variations in Gene Expression and Cell Composition across Lichen Planus Subtypes
Source: Int J Mol Sci. 2024 Sep 8;25(17):9720. doi: 10.3390/ijms25179720 (PMC11396712; doi:10.3390/ijms25179720)
Supplement: Supplementary file 1 [file ijms-25-09720-s001.zip › ijms-3163656-supplementary.pdf]

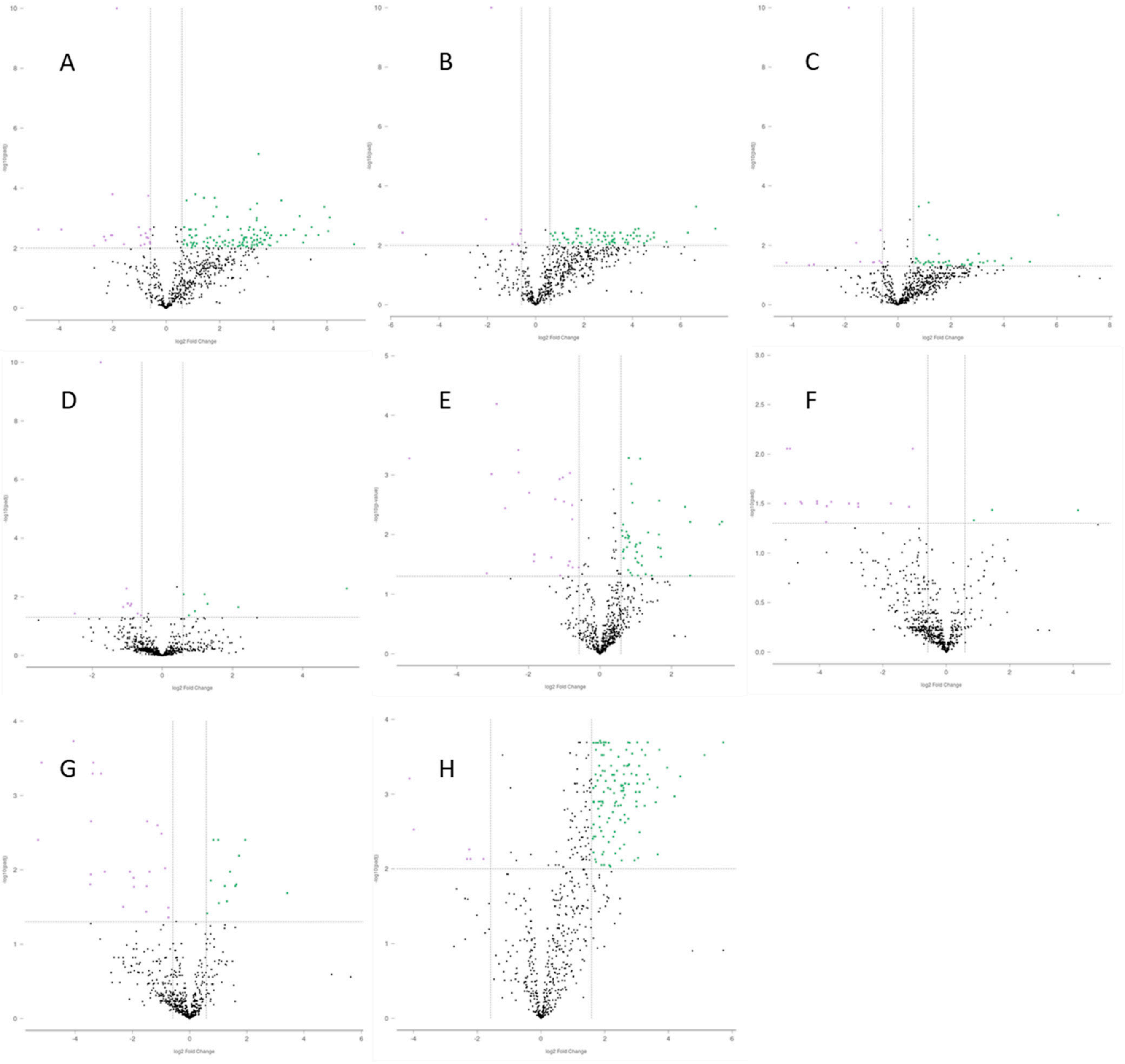

Supplementary Figure S1. Strong differences in gene expression across lichen planus subtypes and healthy skin. NanoString technology was applied to compare the expression of 730 inflammation-related genes across lesional skin samples from 5 classical lichen planus (LP), 4 genital LP, 3 oral LP, 5 lichen planopilaris patients, and 3 healthy skin (NDC) samples. Volcano plots showing differences in expression between the groups of oral LP and HS (A), genital LP and HS (B), classical LP and HS (C), lichen planopilaris and HS (D), genital and oral LP (E),classical and genital LP (F), classical and oral LP (G) and classical LP and lichen planopilaris (H). Upregulated genes are shown in Green, downregulated genes in Pink.

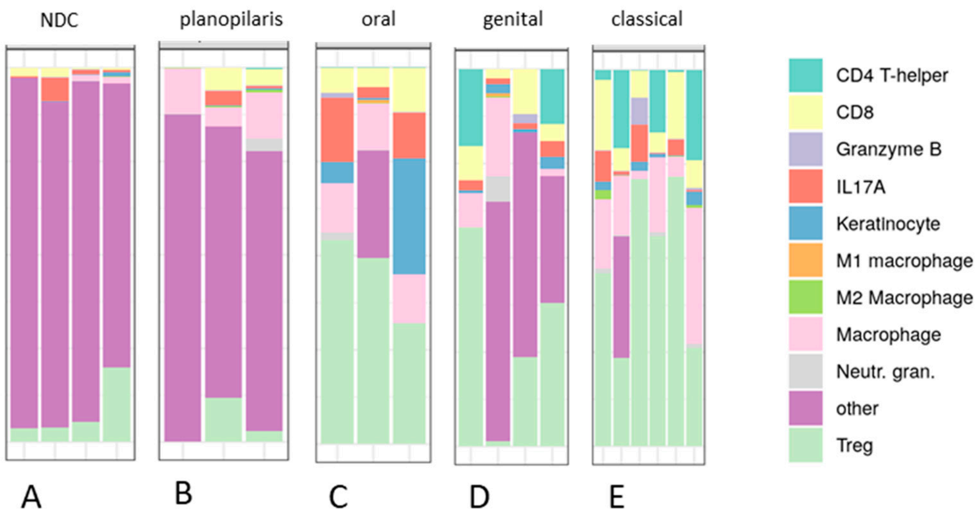

Supplementary Figure S2. Distinct dermal cell-type composition in lichen planus subtypes and healthy skin. Cellular composition among the various lichen planus (LP) subtypes (4 healthy skin (NDC), 6 classical LP, 4 genital LP, 3 oral LP, 3 lichen planopilaris was examined by multiplex immunohistochemistry using the Akoya system, with two panels specifically targeting T cells and innate immune cells. Graph showing the cell-type composition of each patient group individually: NDC (A) lichen planopilaris (B) oral (C) genital (D) classical (E).

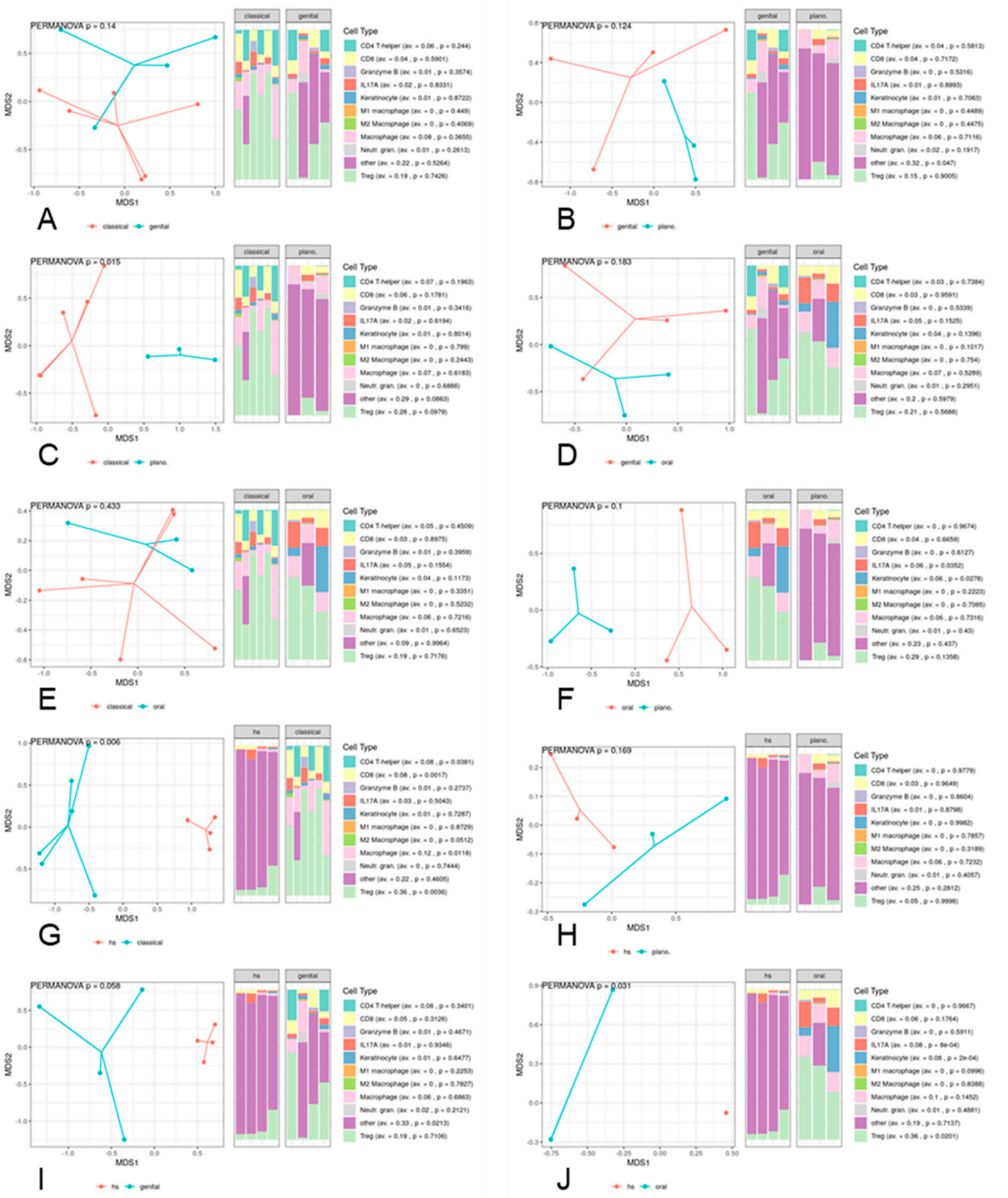

Supplementary Figure S3. Multiplex immunohistochemical analysis shows different cell compositions among lichen planus subtypes. Cellular composition among different subtypes of lichen planus (LP) was investigated by multiplex immunohistochemistry using the Akoya system. The frequency of CD4+ T cells, CD8+ T cells, regulatory T cells (Tregs), cytotoxic cells (Granzyme B+), Th17 cells (IL-17A), keratinocytes, M0, M1 and M2 macrophages, neutrophilic granulocytes and other cells was assessed. The non-metric multidimensional scaling (NMDS) visualization and the cell-type compositions highlight the intrinsic differences in cell composition between the various subtypes of LP (A: classical vs. genital LP, B: genital vs. scalp LP, C: classical vs. lichen planopilaris, D: genital vs. oral LP, E: classical vs oral LP, F: oral vs. lichen planopilaris, G: NDC vs. classical LP, H: NDC vs. lichen planopilairs, I: NDC

vs. genital LP, J: NDC vs. oral LP. The simpler results in parentheses show ("av.") the average contribution of each cell type to the between-groups dissimilarities, on a scale from 0 to 1. This is the magnitude of the contribution. The p-value indicates the consistency of this contribution.

Supplementary Table S1. Prominent pathways in different Lichen planus subtypes.

Term	Significance Score
**A:** Comparison of oral LP and NDC	
Cytotoxicity	3.59
Complement	3.48
CT Antigen	3.43
NK Cell Functions	3.16
Regulation	3.13
T-Cell Functions	3.10
Cell Functions	3.03
Interleukins	2.93
Pathogen Defense	2.86
Cytokines	2.81
**B:** Comparison of genital LP and NDC	
B-Cell Functions	3.21
CT Antigen	3.21
Regulation	3.11
Cytotoxicity	3.05
Adhesion	2.97
Microglial Functions	2.97
TLR	2.96
NK Cell Functions	2.92
T-Cell Functions	2.90
Pathogen Defense	2.85
**C:** Comparison of classical LP and NDC	
CT Antigen	2.81
Cytotoxicity	2.66

Regulation	2.55
T-Cell Functions	2.35
Senescence	2.35
Pathogen Defense	2.33
Transporter Functions	2.29
T-Cell Functions	2.08
NK Cell Functions	2.13
Pathogen Defense	2.85
**D:** Comparison of Lichen planopilairs and NDC	
CT Antigen	2.95
Regulation	2.17
Senescence	1.86
Antigen Processing	1.84
TLR	1.64
Complement	1.60
Transporter Functions	1.59
Cell Cycle	1.58
Chemokines	1.57
**E:** Comparison of genital LP and oral LP	
CT Antigen	1.96
Cell Cycle	1.73
Adhesion	1.71
Transporter Functions	1.62
Interleukins	1.60
Macrophage Functions	1.60
Pathogen Defense	1.57
Cytokines	1.53
Microglial Functions	1.51
Leucocyte Functions	1.36
**F:** Comparison of classical LP and genital LP	
TLR	2.04
Microglial Functions	1.93

Adhesion	1.91
B-Cell Functions	1.82
Chemokines	1.79
Pathogen Defense	1.78
Regulation	1.71
Macrophage Functions	1.63
TNF Superfamily	1.57
Cell Functions	1.52
**G:** Comparison of classical LP and oral LP	
Interleukins	2.45
Pathogen Defense	2.26
Macrophage Functions	2.00
Cytokines	1.96
Transporter Functions	1.90
Cell Functions	1.83
Regulation	1.68
Senescence	1.59
Complement	1.56
Cell Cycle	1.55
**H:** Comparison of classical LP and lichen planopilarisP	
Cytotoxicity	4.52
Antigen Processing	4.35
T-Cell Functions	3.76
B-Cell Functions	3.60
NK Cell Functions	3.58
Pathogen Defense	3.47
Leukocyte Functions	3.45
Regulation	3.36
Cytokines	3.14
Transporter Functions	3.06
